# Does landscape connectivity shape local and global social network structure in white-tailed deer?

**DOI:** 10.1371/journal.pone.0173570

**Published:** 2017-03-17

**Authors:** Erin L. Koen, Marie I. Tosa, Clayton K. Nielsen, Eric M. Schauber

**Affiliations:** 1 Cooperative Wildlife Research Laboratory, Southern Illinois University, Carbondale, Illinois, United States of America; 2 Department of Zoology and Center for Ecology, Southern Illinois University, Carbondale, Illinois, United States of America; 3 Department of Forestry, Southern Illinois University, Carbondale, Illinois, United States of America; Centre for Cellular and Molecular Biology, INDIA

## Abstract

Intraspecific social behavior can be influenced by both intrinsic and extrinsic factors. While much research has focused on how characteristics of individuals influence their roles in social networks, we were interested in the role that landscape structure plays in animal sociality at both individual (local) and population (global) levels. We used female white-tailed deer (*Odocoileus virginianus*) in Illinois, USA, to investigate the potential effect of landscape on social network structure by weighting the edges of seasonal social networks with association rate (based on proximity inferred from GPS collar data). At the local level, we found that sociality among female deer in neighboring social groups (n = 36) was mainly explained by their home range overlap, with two exceptions: 1) during fawning in an area of mixed forest and grassland, deer whose home ranges had low forest connectivity were more social than expected; and 2) during the rut in an area of intensive agriculture, deer inhabiting home ranges with high amount and connectedness of agriculture were more social than expected. At the global scale, we found that deer populations (n = 7) in areas with highly connected forest-agriculture edge, a high proportion of agriculture, and a low proportion of forest tended to have higher weighted network closeness, although low sample size precluded statistical significance. This result implies that infectious disease could spread faster in deer populations inhabiting such landscapes. Our work advances the general understanding of animal social networks, demonstrating how landscape features can underlie differences in social behavior both within and among wildlife social networks.

## Introduction

Social behavior can provide ecological and evolutionary benefits to animals at both individual and population levels. Interactions among individuals can shape individual fitness, with consequent impacts on population demography and structure [1]. For example, individuals that are more social tend to be in better body condition (e.g., African elephants (*Loxodonta africana*) [2]), experience higher pairing success (e.g., male house finches (*Carpodacus mexicanus*) [3]), have higher survival (bottlenose dolphin (*Tursiops* sp.) [4]) and reproductive success (e.g., long-tailed manakin (*Chiroxiphia linearis*) [5], feral horses (*Equus caballus*) [6], bighorn sheep (*Ovis canadensis*) [7]), and have offspring with higher survival rates (e.g., savannah baboons (*Papio cynocephalus*) [8]). Social behavior can also be costly. For example, the social transmission of parasites and infectious diseases can impair host survival and reproduction [9,10]. The evolution and maintenance of sociality is likely the result of species-specific and habitat-specific trade-offs between the costs and benefits of being social [11,12].

Intraspecific social interactions tend to be highly heterogeneous, and that heterogeneity can be driven by a combination of intrinsic and extrinsic factors. Intrinsically, social structure can vary as a function of relatedness (e.g., [13,14]), personality (e.g., [15–17]), and by internal conditions such as reproductive state (e.g., [12]). Indeed, Brent et al.’s [18] findings suggest a genetic basis of sociality in rhesus macaques (*Macaca mulatta*). Extrinsically, both ecological processes and the physical landscape can shape the social structure of populations. Sociality can be influenced by ecological processes such as conspecific population density [19,20], predation risk [21], and hunting pressure [22,23]. The physical landscape can also influence social structure: raccoons (*Procyon lotor*) encountered one another more often when ambient temperature was low [13], African buffalo (*Syncerus caffer*) herds were less clustered during a drought [24], oribi (*Ourebia ourebi*) groups were larger when forage abundance was high [25], and the frequency of associations in mixed-species bird flocks declined with increasing habitat fragmentation [26]. Nonetheless, identifying the mechanisms underlying the structure of social networks, such as the influences of environment and habitat, remains an understudied aspect of social network ecology [1].

We investigated the influence of extrinsic landscape features on the social network structure of female white-tailed deer (*Odocoileus virginianus*). Female white-tailed deer represent an interesting model to explore social behavior because they form matrilineal social groups [27]. These groups show strong associations in space and time among females and male fawns within a group and relatively few associations among individuals in neighboring groups [28,29]. Contact among neighboring groups of female white-tailed deer does occur, however, and could be a main conduit of disease spread across larger spatial scales (e.g., [30]). Our objective was to assess the role of landscape structure in shaping sociality among social groups of deer at both individual (local) and population (global) levels. In winter, associations among deer tend to be low in forested areas relative to non-forested areas, as home ranges tend to be smaller and overlap less with those of neighbors, and group sizes tend to be smaller [31]. Thus, we expected that the amount of forest would influence the structure of social networks: deer whose home ranges had low forest amount would have higher node centrality (i.e., would be more central in a network). There is also evidence suggesting that landscape connectivity influences disease risk in white-tailed deer, presumably by impacting host movements [32]. Assuming that connected landscapes facilitate deer movement and thus increase the likelihood of encountering neighbors, we also expected that landscape connectivity would influence the structure of social networks: deer whose home ranges had highly connected forest or edge would have higher node centrality. Finally, we expected these patterns to translate to global social network structure, such that average weighted network closeness (i.e., the average weighted distance of the shortest path between one node and all other nodes in the network) would be higher in areas of low forest amount and high connectivity of forest or edge. Not only does our study address how landscape features influence individual- and population- (i.e., network) level social behavior, it will also have implications for disease transmission within and among populations.

## Methods

### Study areas

**Fig 1:**
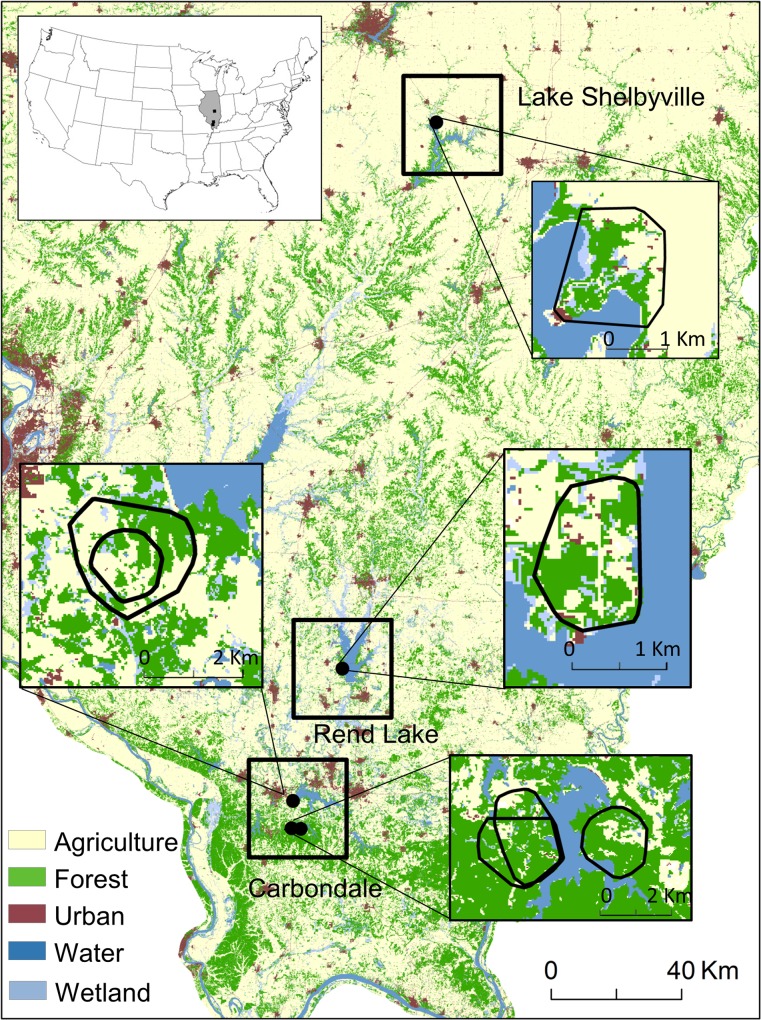
Five study areas (black dots) within 3 regions (black squares) in southern Illinois, USA. Inset maps show minimum convex polygons (MCP) around the locations of all monitored female white-tailed deer (*Odocoileus virginianus*) for each study area during the gestation period (1 Jan– 10 Mar), as well as the location of Illinois (grey) within USA. Within the Carbondale region, the upper dot represents the Carbondale study area (inner and outer MCPs are Carbondale 2012 and 2005, respectively), the lower left dot represents the Touch of Nature study area (2012 and 2013 MCPs at Touch of Nature overlap), and the lower right dot represents the Crab Orchard study area. Land cover data are from the Illinois Natural History Survey Illinois Gap Analysis Land Cover Classification from 1999 and 2000 [33]. The forest category includes dry, mesic, and dry-mesic upland forest and mesic and wet-mesic floodplain forest. The agriculture category consists of soy, corn, rural grassland (permanent pastureland, roadsides and fence lines, railroad right-of-ways, waterways, prairies, and other grassland cover), winter wheat, and other small grains and hay. Water represents lakes and rivers, and wetlands includes both treed and untreed wetlands.

We conducted our study within 3 regions: Rend Lake and Carbondale in southern Illinois, and Lake Shelbyville in central Illinois, USA (Fig 1). Within the Carbondale region, we defined 3 study areas: “Carbondale”, “Crab Orchard”, and “Touch of Nature”. We obtained land cover data from the Illinois Natural History Survey Illinois Gap Analysis Land Cover Classification [33]. These data were derived from 1999 and 2000 Landsat 5 and Landsat 7 Thematic Mapper satellite imagery with a ground resolution of 30 m by 30 m. Land cover composition of our study areas (S1 Table) ranged from forest-dominated Crab Orchard and Touch of Nature (64% and 66% forest, and 14% and 15% agriculture, respectively) to crop-dominated Lake Shelbyville (63% agriculture, mostly corn and soybean, and 12% forest). Carbondale and Rend Lake areas had fairly even mixtures of forest and grassland and a relatively low proportion of cropland. Southern and central Illinois experienced moderate winters from 2002–2014, with mean January lows ranging from -4.0°C (SD 2.8) in Carbondale to -7.0°C (SD 3.3) in Lake Shelbyville, and mean July highs ranging from of 31.4°C (SD 2.2) in Carbondale to 30.2°C (SD 2.3) in Lake Shelbyville [34]. Chronic wasting disease, a transmissible spongiform encephalopathy, has been detected in white-tailed deer in Wisconsin and northern Illinois, but not yet in central and southern Illinois, where our study took place [35].

#### Telemetry datasets

We used published GPS telemetry datasets [28,29,36] from female white-tailed deer captured in our 5 study areas. The number of deer collared at any one time varied by study area, year, and season (Tables 1 and 2); together, we used GPS data from 24 female white-tailed deer from Carbondale (2002–2006), 6 deer from Carbondale (2012), 12 deer from Lake Shelbyville (2007–2009), 5 deer from Crab Orchard (2014), 4 deer from Rend Lake (2014), and 18 deer from Touch of Nature (2012–2013), for a total of 69 female deer. Deer capture protocols are described in Schauber et al. [28,36] and Tosa et al. [29]. The U.S. Army Corps of Engineers and the Illinois Department of Natural Resources (IDNR: Lake Shelbyville), the IDNR (Rend Lake), the US Fish and Wildlife Service (Crab Orchard), Southern Illinois University Carbondale (Touch of Nature), and private landowners granted us permission to conduct our field studies on their land. Our study did not involve endangered or protected species. Southern Illinois University Carbondale’s Institutional Animal Care and Use Committee approved our capture and handling methods (protocols 03–003 and 11–027). The estimated population density of deer was similar in Lake Shelbyville (11–18 deer/km^2^) and near the Carbondale site (15–19 deer/km^2^; [37]). The median position error of stationary collars in closed canopy was 8.8m [36]. We were interested in social interactions among deer social groups, thus we omitted deer that belonged to the same social group as one other collared deer (identified by Schauber et al. [28] and Tosa et al. [29] based on correlated movement patterns and consistent proximity; see [28,29] for details). To make our datasets comparable, for each deer we retained locations obtained every 2 hours.

**Table 1:** The number of female white-tailed deer (*Odocoileus virginianus*) with >600 GPS locations within 3 seasons^a^ used in our local network analysis and the mean number of simultaneous locations^b^ across dyads.

Study area	Year	Season	No. deer	Simultaneous locations	
				Mean	SD
Carbondale	2002	Rut	3	699.7	39.4
	2003	Gestation	6	741.1	129.8
		Rut	3	852.0	112.6
	2004	Gestation	11	1071.3	237.5
		Fawning	4	1155.0	27.6
		Rut	7	950.4	358.7
	2005	Gestation	7	1406.9	112.8
		Fawning	7	1099.7	52.0
		Rut	6	1176.4	255.0
	2006	Gestation	5	676.8	14.4
Lake Shelbyville	2007	Gestation	6	1168.7	62.6
		Fawning	4	957.0	305.5
		Rut	2	1381.0	0.0
	2008	Gestation	10	994.0	394.4
		Fawning	6	1196.6	22.4
		Rut	4	1390.5	13.7

^a^ Gestation (1 Jan– 14 May), fawning (15 May– 31 Aug), rut (1 Sep– 31 Dec)

^b^ Locations obtained within 3 minutes

**Table 2:** Datasets used in our global analysis of female white-tailed deer (*Odocoileus virginianus*) network^a^ structure during the gestation period^b^.

Study area	Year	No. deer	Simultaneous locations^c^		Network closeness^d^		
			Mean	SD	Mean	SD	No. combinations
Carbondale	2005	6	740.8	72.3	2.81	0.21	15
	2012	6	832.4	2.4	3.00	0	15
Lake Shelbyville	2009	4	810.3	4.8	3.00	NA	1
Crab Orchard	2014	5	798.3	6.2	2.87	0.12	5
Touch of Nature	2012	13	804.1	39.0	2.09	0.74	715
	2013	5	719.9	65.4	2.39	0.55	5
Rend Lake	2014	4	716.3	90.6	3.17	NA	1

^a^ We weighted network edges with the residuals of the linear relationship between association rate (the number of times two deer were with 25m of one another at the same time divided by the total number of simultaneous locations) and log home range overlap (volume of intersection).

^b^ 1 Jan– 10 Mar.

^c^ Mean number of simultaneous (within 3 minutes) locations across dyads.

^d^ To compare network closeness among networks of different sizes, we subsampled our networks such that they contained 4 nodes. We calculated the average weighted closeness for all unique 4-node combinations of the original set of nodes. For example, the Carbondale 2012 study area had 6 nodes–we calculated the average weighted closeness across all 15 possible 4-node combinations.

### Association rate

We used the packages wildlifeDI [38] and adehabitatLT [39] in R [40] to calculate association rate: the number of times that 2 deer were within 25m of one another at the same time (within 3 minutes), divided by the number of simultaneous locations obtained for that dyad. Social behavior of female deer tends to vary by season [28,36], thus we defined 3 seasons based on Schauber et al. [28]: gestation (Jan 1 –May 14), when female white-tailed deer occur in matrilineal social groups and larger groups of deer tend to aggregate to feed [27], fawning (May 15 –Aug 31), when adult female white-tailed deer tend to be solitary [27,41,42], and the rut (Sep 1 –Dec 31), when female white-tailed deer tend to re-establish matrilineal social groups [43].

Including only those dyads that were collared simultaneously for an entire season would have required that we censor a large proportion of our data. Instead, we assumed that deer behavior during a portion of the season was representative of the entire season. We tested this assumption by assessing whether association rates during particular months were biased relative to the seasonal association rate. We also estimated how many simultaneous locations were necessary for accurate estimates of seasonal association rates. We present these analyses in S1 Appendix and based on the results, we did not include deer dyads with <600 simultaneous locations per season (S1 Fig) or dyads that were monitored only in Apr-May or Jun-Jul (S2 Fig), as association rates during these months were likely biased low relative to the entire season. We included only dyads with seasonal home ranges that overlapped, thus omitting pairs of deer that did not interact because they were not collared at the same time or because their home ranges were not neighboring. Because we subsampled our data to include only dyads with sufficient data, our sample was not truly random.

### Amount and connectivity of landcover

#### Amount

We created three binary grids with 30m x 30m cells representing the presence of forest (upland and floodplain forests), agriculture (corn, soybean, winter wheat, rural grassland, and other small grains), and forest-agriculture edge [33]. We used Geospatial Modelling Environment software (version 0.7.2.0; [44]) to find the edges between agriculture and forest and then coded 30m x 30m pixels based on presence or absence of edge.

#### Connectivity

We used circuit theory [45] with software Circuitscape 4.0 [46] to estimate connectivity of the landscape for deer with respect to forest, agriculture, and edge. Circuit theory models animal movement across a landscape using the analogous properties of random walk and electricity moving through a circuit [47]. Electric current moves across the conductance surface between pairs of populations (or sites), such that multiple or wider swaths of suitable habitat are conduits of current, or correspondingly, animal movement, relative to narrow pathways [48]. The resultant map of current density is meant to represent a prediction of functional connectivity, with high pixel values representing a high probability of use by random walkers.

We wanted to model functional connectivity of the landscape in all directions, rather than between pairs of sites. Thus, we used the method described by Koen et al. [49] whereby we placed sites, representing the start and end points for current to be shunted through the circuit, around the outside of our study areas (S3 Fig). We converted binary grids for each land cover type (forest, agriculture, or edge) into conductance surfaces by replacing each "1" pixel value (presence) with 101 and each "0" pixel value with 1. In this way, we created 3 conductance maps meant to represent the ease of movement through types of landcover, with high values assigned to the presence of potentially suitable landcover types (S3 Fig). We defined our three study regions as 30km x 30km areas (Fig 1) and we placed a 7.5km-wide buffer around each study region; these regions were sufficiently large such that they did not influence our estimates of current density [49]. We then placed 50 sites at equal intervals (every 3.6 km) around the perimeter of each buffer and used Circuitscape to model the connectivity of each conductance surface between those sites (S3 Fig). We clipped the resultant current density maps by the width of the buffer to remove pixels that might be biased high because of the arbitrary placement of the 50 sites [49] (S4 Fig).

### Local and global network structure

We assessed the influence of amount and connectivity of landcover on deer sociality at both individual (local) and population (global) levels. We defined a population (or network) loosely as a group of monitored deer in a spatially and temporally defined area such that monitored individuals could potentially interact. A population was made up of several monitored deer, and each monitored deer represented one member of a social group. We constructed social networks for each study area, season, and year with the tnet package [50] in R, with nodes representing individual deer and edges representing social interaction, with edges weighted by association rate. We used the term dyad to represent a pair of deer that could be connected by an edge (i.e., two deer from the same study area with >600 simultaneous locations).

#### Local network analysis

Here, we used network attributes at the individual (node) level as an index of deer sociality. We used GPS data for deer monitored at the Carbondale (2002–06; n = 24) and the Lake Shelbyville (2007–09; n = 12) study areas only because we had data for all three seasons (Table 1). When an individual deer was collared for >1 year, we included that individual only once in each season by omitting the season-year with the fewest GPS locations. We sought a network metric to represent deer sociality at the node level that would be relatively insensitive to the likelihood that not all of the neighboring deer were simultaneously monitored. Both degree (number of edges connected to a node) and weighted degree (the sum of the edge weights connected to a node) should depend on the proportion of neighboring deer that were monitored. Likewise, betweenness centrality (the number of times the shortest path through the network goes through a particular node) should also depend on the number of neighboring deer that were monitored. Instead, we used the average weighted degree (sum of edge weights divided by the number of edges at each node) as our measure of node-based sociality because it should be less sensitive to the proportion of monitored neighbors. We included all potential edges in our estimate of average edge weight (i.e., edges with a weight of zero: these edges represented simultaneously monitored dyads that shared space but not necessarily at the same time). We assigned these edges a weight of 1x10^-5^, a value less than the lowest association rate that we could expect (0.00062; S1 Appendix). Seasonal networks for Carbondale and Lake Shelbyville had an average of 5.9 (SD = 2.4, range = 3–11) and 5.4 (SD = 2.5, range = 2–10) collared deer, respectively (Table 1).

#### Local independent variables

We calculated seasonal 95% kernel density home ranges [51] using a reference bandwidth (href) with adehabitatHR [39] in R (S2 Table). We estimated seasonal home range overlap for deer dyads during the time that they were both monitored as the probability of animal *i* being in animal *j*’s home range (PHR; [52]). This measure differed from association rate in that it was static; it did not require that animals use the same space at the same time. We used PHR because we were interested in quantifying the amount of space that each individual shared with all of its neighbours. Thus for each individual, we calculated the average home range overlap with neighboring collared deer.

For each deer, we calculated the proportion of forest, agriculture, and edge within its seasonal home range (95% kernel density contour). We also calculated the average current density, our index of landscape connectivity, based on forest, agriculture, or edge, within each deer’s seasonal home range.

#### Local models

We created models (S2 Appendix) to describe the influence of extrinsic landscape features on social network structure at the level of the individual node. For each study area (Carbondale and Lake Shelbyville), we used linear regression to identify the relationship between at-site deer sociality (i.e., average edge weight of nodes, with edges weighted by association rate) and our independent variables (i.e., home range overlap and both proportion and connectivity of forest, agriculture, and edge). We used a Box-Cox power transformation on the dependent variable (λ = 0.22; [53]) with the R package MASS (7.3–45; [54]) because the relationship between average edge weight and home range overlap was heteroscedastic (S5 Fig). We used information-theoretic model selection (AIC_c_; [55]) with the package MuMIn (ver. 1.13.4; [56]) in R to identify top models (Δ AIC_c_ <2) and estimate model averaged coefficients. The proportion of agriculture was negatively correlated with the proportion of forest and positively correlated with the connectivity of agriculture, forest connectivity was negatively correlated with agriculture connectivity, and edge connectivity was positively correlated with forest connectivity and the proportion of edge (Pearson *r* >|0.5|; S3 Appendix). Thus, we included only one landscape variable in each model. We pooled data over years and modeled each study area and season separately to account for differences among regions and seasons. We used permutation tests to assess whether our top models were different than what we would expect by chance; we permuted the dependent variable 9999 times and considered whether the observed test statistic from the real data was larger in magnitude than values derived from 95% of the permutations.

#### Global network analysis

To assess the influence of amount and connectivity of landcover on social connectedness at the population level, we used individual networks as our sample unit. We included 7 networks from 5 study areas that had >4 unique nodes with association rate >0 in the gestation period: Carbondale 2005 and 2012, Lake Shelbyville 2009, Crab Orchard 2014, Touch of Nature 2012 and 2013, and Rend Lake 2014 (Table 2). We subset the GPS locations to include 1 Jan– 10 Mar only because after 10 Mar, baiting and sharpshooting of deer occurred in the Crab Orchard, Touch of Nature, Rend Lake, and Carbondale 2012 study areas. We included only dyads with >600 simultaneous locations and >0 home range overlap.

We were unable to include >1 independent variable in our models because we had a sample size of 7 networks. Thus, to control for the relationship between home range overlap and association rate (S6 Fig), we weighted network edges with the standardized residuals of a linear regression fit to the relationship between home range overlap and (log) seasonal association rate for each dyad (pooled over all 7 networks). To estimate home range overlap, we used the volume of intersection (VI) of 95% kernel utilization distributions (UD; [52]) for each dyad during the time that both individuals were monitored. This estimate ranged from 0 (no overlap) to 1 (identical UDs). We used VI because we were interested in quantifying the space shared by the dyad.

From these edge weight data, we calculated weighted network closeness [57,58] as our dependent variable with the tnet package [50] in R as per Opsahl et al. [58]:

$$closeness(i)=\sum_{j}\left(\frac{1}{d_{ij}}\right),$$

where *i* is the focal node, *j* represents another node in the network, and *d*_*ij*_ is the shortest weighted path through the network between *i* and *j* based on Dijkstra’s [59] algorithm [58]. For each network, we found the average weighted closeness across all nodes. Network closeness, however, is dependent on the number of nodes in the network: the shortest weighted path between any 2 nodes has the potential to be shorter when there are fewer nodes. To compare network closeness among networks of different sizes, we subsampled our networks such that each subsample contained 4 nodes. We calculated the average weighted closeness for all 4-node combinations, and we used this average value as our dependent variable. For example, the Carbondale 2005 network had 6 nodes; we calculated average closeness for all 15 unique 4-node combinations from the set of 6 nodes. If the removal of node *j* resulted in node *i* being isolated from the network, node *i* contributed a value of 0 to the average. We did not replace zero-weighted edges with 1x10^-5^ as we did in the local-scale analysis; our estimates of closeness were based on the shortest weighted path through the network, and including an edge for dyads that shared space but not at the same time (i.e., >0 home range overlap but 0 association rate) might have influenced the shortest path.

#### Global independent variables and models

For each network, we defined a study area with a 100% minimum convex polygon [60] around all GPS locations. We then calculated the proportion of forest, agriculture, and edge within each study area, as well as the average current density related to each of these variables. Because all competing models had an equal number of parameters, we simply compared the variance explained (R^2^) by univariate linear regression models describing the relationship between average weighted network closeness and each landcover proportion and connectivity variable.

## Results

### Influence of landscape on local network connectivity

The degree of home range overlap was the best predictor of sociality among deer groups: across seasons and sites, deer that shared more space tended to have higher association rates (Tables 3 and 4). In analyzing association rates during gestation in the Carbondale dataset, models that included home range overlap with forest connectivity, edge connectivity, or edge proportion had F-statistics >95% of randomized values (Table 3). However, the confidence intervals of the coefficients of these variables alone overlapped zero (S4 Table), indicating that home range overlap had the greatest influence on deer sociality during gestation in both Carbondale and Shelbyville (Table 4). During fawning, when association rates tended to be lower (S3 Table), both the connectivity of forest and home range overlap best predicted deer sociality in Carbondale (Table 3): female deer in highly connected forest tended to be less social with their neighbors (S4 Table; note that we did not find an effect of landcover or home range overlap on deer sociality in Lake Shelbyville during fawning (Table 4)). During the rut, the best predictors of high sociality among deer groups in Lake Shelbyville were high amount and high connectivity of agricultural land, and not home range overlap (Table 4, S5 Table). Although the 95% CI of the model-averaged coefficients for agriculture amount and connectivity did not overlap zero (S5 Table) and the F-statistics of these models were >95% of randomized values, the null model was competitive in this analysis (Table 4), and therefore evidence for an effect of landscape was weak.

**Table 3:** Top models (Δ AIC_c_ < 8) predicting the average edge weight in seasonal networks of female white-tailed deer (*Odocoileus virginianus*) association rate^a^ in Carbondale, Illinois (2002–2006).

Season^b^	Model^c^	k	AIC_c_	Δ AIC_c_	Weight	R^2^ ^d^
Gestation	**HR overlap [+]**	**3**	**-49.43**	**0**	**0.25**	**0.65**
	**Forest (conn) [–] + HR overlap [+]**	**4**	**-49.06**	**0.37**	**0.21**	**0.69**
	**Edge (conn) [–] + HR overlap [+]**	**4**	**-48.67**	**0.75**	**0.17**	**0.68**
	**Edge (prop) [–] + HR overlap [+]**	**4**	**-47.88**	**1.54**	**0.12**	**0.67**
	Ag (conn) + HR overlap	4	-47.39	2.04	0.09	0.67
	Ag (prop) + HR overlap	4	-47.37	2.06	0.09	0.66
	Forest (prop) + HR overlap	4	-46.85	2.58	0.07	0.66
Fawning	**Forest (conn) [–]**	**3**	**-24.12**	**0**	**0.58**	**0.72**
	**Forest (conn) [–] + HR overlap [+]**	**4**	**-22.20**	**1.92**	**0.22**	**0.79**
	Forest (prop)	3	-20.94	3.17	0.12	0.62
	Ag (conn)	3	-17.75	6.36	0.02	0.50
	Forest (prop) + HR overlap	4	-17.06	7.06	0.02	0.67
	Ag (prop)	3	-16.69	7.42	0.01	0.44
	Ag (prop) + HR overlap	4	-16.57	7.55	0.01	0.65
Rut	**HR overlap [+]**	**3**	**-26.23**	**0**	**0.39**	**0.33**
	Forest (conn) + HR overlap	4	-23.29	2.93	0.09	0.35
	Ag (prop) + HR overlap	4	-23.17	3.06	0.08	0.34
	Ag (conn) + HR overlap	4	-23.06	3.17	0.08	0.34
	Edge (prop) + HR overlap	4	-22.85	3.37	0.07	0.33
	Forest (prop) + HR overlap	4	-22.83	3.40	0.07	0.33
	Edge (conn) + HR overlap	4	-22.75	3.48	0.07	0.33
	Null	2	-22.49	3.74	0.06	
	Ag (conn)	3	-19.89	6.33	0.02	0.02
	Forest (prop)	3	-19.71	6.52	0.01	0.01
	Edge (prop)	3	-19.70	6.53	0.01	0.01
	Forest (conn)	3	-19.67	6.56	0.01	0.01
	Edge (conn)	3	-19.65	6.57	0.01	0.01
	Ag (prop)	3	-19.52	6.71	0.01	1.1 x10^-3^

Models with Δ AIC_c_ < 2 are in bold font.

^a^ We weighted network edges by association rate; the number of times two deer were within 25m of one another at the same time divided by the total number of simultaneous locations.

^b^ Gestation (1 Jan– 14 May; n = 24), fawning (15 May– 31 Aug; n = 11), rut (1 Sep– 31 Dec; n = 17).

^c^ [+] and [–] indicate the direction of each variable’s effect. Ag (prop), forest (prop), and edge (prop) are the proportions of agriculture, forest, and the edge between forest and agriculture, respectively, within seasonal home ranges of deer. Ag (conn), forest (conn), and edge (conn) represent the mean connectivity (current density) of agriculture, forest, and the edge between forest and agriculture, respectively, within seasonal home ranges of deer. HR overlap is the mean probability of a neighboring deer being within an individual’s 95% kernel density home range during the time that each pair of deer was simultaneously monitored (PHR, [52]), averaged over all neighbors for each deer.

^d^ All models with Δ AICc <2 had F-statistics >95% of randomized values.

**Table 4:** Top models (Δ AIC_c_ < 8) predicting the average edge weight in seasonal networks of female white-tailed deer (*Odocoileus virginianus*) association rate^a^ in Lake Shelbyville, Illinois (2007–2009).

Season^b^	Model^c^	k	AIC_c_	Δ AIC_c_	Weight	R^2^ ^d^
Gestation	**HR overlap [+]**	**3**	**-15.27**	**0**	**0.60**	**0.68**
	Ag (prop) + HR overlap	4	-11.29	3.99	0.08	0.70
	Ag (conn) + HR overlap	4	-10.89	4.39	0.07	0.69
	Forest (conn) + HR overlap	4	-10.70	4.58	0.06	0.68
	Forest (prop) + HR overlap	4	-10.66	4.61	0.06	0.68
	Edge (prop) + HR overlap	4	-10.57	4.70	0.06	0.68
	Edge (conn) + HR overlap	4	-10.56	4.71	0.06	0.68
Fawning	**Null**	**2**	**-3.88**	**0**	**0.41**	
	**HR overlap [+]**	**3**	**-2.45**	**1.43**	**0.20**	**0.25**
	Ag (conn)	3	0.09	3.97	0.06	0.03
	Ag (prop)	3	0.25	4.13	0.05	0.02
	Edge (prop)	3	0.30	4.18	0.05	0.01
	Forest (prop)	3	0.30	4.18	0.05	0.01
	Edge (conn)	3	0.37	4.25	0.05	3.5 x10^-3^
	Forest (conn)	3	0.40	4.28	0.05	8.5 x10^-4^
	Forest (prop) + HR overlap	4	2.04	5.92	0.02	0.35
	Ag (prop) + HR overlap	4	2.66	6.53	0.02	0.31
	Forest (conn) + HR overlap	4	2.67	6.55	0.02	0.31
	Edge (conn) + HR overlap	4	2.70	6.58	0.02	0.31
	Ag (conn) + HR overlap	4	2.94	6.82	0.01	0.29
	Edge (prop) + HR overlap	4	3.09	6.97	0.01	0.28
Rut	**Ag (prop) [+]**	**3**	**-4.98**	**0**	**0.40**	**0.82**
	**Null**	**2**	**-4.63**	**0.36**	**0.33**	
	**Ag (conn) [+]**	**3**	**-3.86**	**1.12**	**0.23**	**0.78**
	Edge (conn)	3	1.55	6.54	0.02	0.47
	Edge (prop)	3	2.85	7.84	0.01	0.34
	Forest (conn)	3	3.02	8.00	0.01	0.32

Models with Δ AIC_c_ < 2 are in bold font.

^a^ We weighted network edges by association rate; the number of times two deer were within 25m of one another at the same time divided by the total number of simultaneous locations.

^b^ Gestation (1 Jan– 14 May; n = 12), fawning (15 May– 31 Aug; n = 10), rut (1 Sep– 31 Dec; n = 6).

^c^ [+] and [–] indicate the direction of each variable’s effect. Variables are described in the footnote of Table 3.

^d^ All models with Δ AICc <2 had F-statistics >95% of randomized values except the home range overlap model during fawning.

### Influence of landscape structure on global network closeness

**Fig 2:**
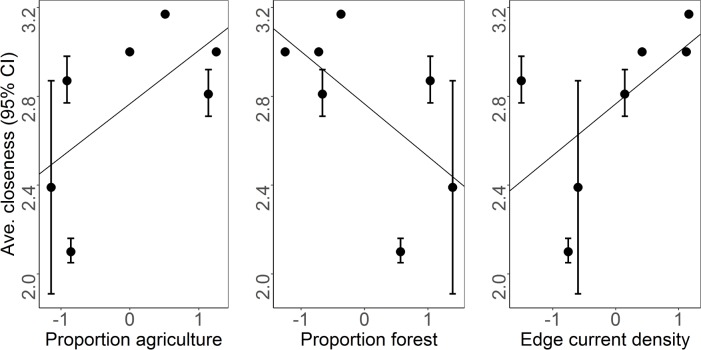
The relationship between average weighted network closeness of 7 global social networks of female white-tailed deer (*Odocoileus virginianus*) in Illinois, USA and the average standardized (z-score) proportion of agriculture, the average standardized proportion of forest, and the average standardized current density of forest-agriculture edge within a 100% MCP around all deer GPS locations for each population.

Accounting for home range overlap and the number of nodes in the network, estimates of weighted network closeness during the early gestation period ranged from 2.09 (Touch of Nature 2012) to 3.17 (Rend Lake; Table 2). We found relationships between mean weighted network closeness and the amount of agriculture and forest: deer populations in areas with high amounts of agriculture and low amounts of forest tended to be more socially connected (R^2^ = 0.40 and R^2^ = 0.39, respectively; Fig 2, Table 5), although the small number of networks we analyzed meant that these results did not reach statistical significance. We also found that deer populations in areas with highly connected edge tended to be more socially connected (R^2^ = 0.38; Fig 2, Table 5).

**Table 5:** Standardized coefficients and fit of univariate linear models predicting global weighted network closeness. Networks (n = 7) represent female white-tailed deer (*Odocoileus virginianus*) association rate^a^ during the early gestation period^b^ in central and southern Illinois, USA.

Predictor variable^c^	Standardized coefficient	SE	R^2^	P
Ag (prop)	0.242	0.131	0.405	0.124
Forest (prop)	-0.239	0.133	0.394	0.131
Edge (conn)	0.235	0.134	0.381	0.140
Edge (prop)	0.146	0.157	0.148	0.395
Forest (conn)	0.124	0.161	0.105	0.477
Ag (conn)	0.057	0.168	0.023	0.747

^a^ We weighted network edges with the standardized residuals of a linear model fit to the relationship between home range overlap and log seasonal association rate (the number of times two deer were within 25m of one another at the same time, divided by the number of simultaneous locations).

^b^ 1 Jan– 10 Mar (2005, 2012 in Carbondale; 2009 in Lake Shelbyville; 2012, 2013 in Touch of Nature; 2014 in Crab Orchard and Rend Lake (Fig 1)).

^c^ Variables are described in the footnote of Table 3.

## Discussion

Social network structure can provide accurate predictions of animal movement across landscapes [61]. We explored the influence of an extrinsic factor, landcover composition, on female white-tailed deer social network structure and found that landscape structure can impact sociality at the population level, and to a lesser extent, at the individual level. Indeed, there is a movement within social network ecology to move beyond descriptive network statistics and identify the ecological processes underlying non-random social structure [1]. Our findings add to our current knowledge of mechanisms that can shape social networks.

At an individual level, we generally found that deer sociality (i.e., centrality derived from proximal associations) was highly correlated with home range overlap and not with the landscape variables that we measured. Our finding that landscape connectivity had little influence on deer movement could suggest that deer movement was not impeded by the agricultural matrix. We found two exceptions, however. First, during fawning we found that where forest and grassland were relatively abundant (i.e., Carbondale), forest connectivity and home range overlap were the best predictors of sociality among groups; deer occupying home ranges with low forest connectivity tended to be more social. The connectivity of forest in our study appeared greatest where there were corridors of forest surrounded by non-forest, termed “pinch points” [45] (S7 Fig). Although deer are generally less social during fawning [27,36], corridors of connected forest appeared to impede social behavior; deer appeared to be more social where forest tracts were wider—pixels in these wider tracts of forest tended to have a lower probability of movement (i.e., lower current density). Second, during the rut we found that where row crops were relatively abundant (i.e., Lake Shelbyville), both the amount and connectivity of agriculture, independent of home range overlap, were the best predictors of sociality among groups. Kjær et al. [42] found that associations among deer tended to occur where deer feed or take cover, and Walter et al. [62] found that during the non-growing season, white-tailed deer moved away from forest cover in search of suitable forage. We speculate that in the Lake Shelbyville study area, the presence of waste crops and limited forest browse resulted in increased sociality among groups in agricultural areas.

Hawkins and Klimstra [27] noted that deer in southern Illinois tended to aggregate in large herds (25–30 individuals) in late winter and early spring; these herds were temporary and consisted of several social groups. An alternate driver of the relationship between landscape structure and associations among deer could be landcover complexity leading to the aggregation of social groups. Herd size for many large herbivores tends to increase with landscape openness (e.g., [63–65]). Indeed, both Hirth [66] and Habib et al. [31] observed that within a population of white-tailed deer, group size tended to be smaller in dense cover and larger in open landscapes. This pattern could be an adaptation to predation pressure: in dense cover, an individual can better avoid being detected by a predator in a small herd, whereas in open landscapes, the benefits of belonging to a large group include group vigilance and dilution of predation [67,68]. This pattern could also be related to the idea that individuals in open landscapes are simply more likely to detect one another and form larger herds [69]. Finally, this pattern could be a function of resource distribution (e.g., [70]). We found that during the rut in an area with abundant row crops, the best predictors of sociality among social groups were the amount and connectivity of agriculture. It is possible that sociality among deer social groups is related to larger herd size in agricultural areas (note that we do not have data on herd size) or to a combination of herd size and the relative ease of movement through connected agricultural land.

We expected that deer in less connected landcover would be less social because their movements would be impeded; this expectation assumes that there is otherwise little inter-individual variation in an individual’s social behavior toward neighboring groups. For many species, genetic relatedness plays a role in determining social behavior (e.g., hyenas (*Crocuta crocuta*) [71], wild boars (*Sus scrofa*) [14], and barnacle geese (*Branta leucopsis*) [72]). There are also several species for which this relationship has not been shown (e.g., southern flying squirrels (*Glaucomys volans*) [73], elk (*Cervus canadensis*) [74], and raccoons [75]). Association rates among female white-tailed deer in the same social group tend to be higher than among deer in neighboring social groups [28], and individual deer within the same social group or in close spatial proximity tend to be related ([27,76,77], but see [78,79]). Magle et al. [80] found that related white-tailed deer shared more space than unrelated deer. If this was the case for the female deer in our study (note that we do not know the relatedness of our collared deer), then relatedness could be driving some of the patterns that we observed because home range overlap was a component of the majority of the top models predicting individual deer sociality. In our population-level analysis, however, we used the residuals of the relationship between association rate and home range overlap to weight the edges of our network. Thus, landscape connectivity was having some effect on the social structure of deer at the population level, independent of space sharing and possibly genetic relatedness.

At a global level, we found some evidence that network structure was related to landscape structure: network connectivity (i.e., average closeness centrality) was higher in agricultural areas and areas with high connectivity of edge and lower in forested areas. This suggests that landscape features facilitating social behavior at the population level could operate through a combination of the relative ease of movement through connected edge and the effects of open landscapes, such as larger home ranges that tend to overlap more [31] or group aggregation in open agriculture relative to forest [66,69]. We note that there may still be an effect of the number of nodes per network on our estimates of average closeness, despite our efforts to remove its effect: networks with few nodes appeared to have higher estimates of average closeness. Thus, it is unclear whether the number of nodes is somehow influencing the relationship that we observed between average closeness and our landscape variables. The apparent relationship between landscape and social network structure has important implications for disease spread in free-ranging populations of white-tailed deer.

Infectious diseases pose a significant threat to global biodiversity [81]; understanding the biology and ecology of animal sociality that contributes to disease spread is therefore imperative. Chronic wasting disease is a fatal, transmissible spongiform encephalopathy afflicting cervids, including white-tailed deer, and has been spreading across several regions of Canada and the USA [82]. The disease is transmitted both directly through contact by infectious individuals and indirectly through infectious prions in the environment [83,84]. Research has shown that physical contact among female white-tailed deer within the same social group is relatively high [28] and that within-group social interaction is a central route of CWD transmission [76]. Although our study populations were not impacted by CWD, we modeled factors that could influence sociality among groups, which is relevant for inference of disease spread across larger spatial scales. Our local results suggest that overall, deer sociality among groups was not strongly influenced by landscape heterogeneity, except in Carbondale during fawning and Lake Shelbyville during the rut, where landscape structure had the potential to create “super-spreaders” of infectious disease; individuals whose behavior causes them to infect disproportionately more secondary contacts [85,86]. At the population level, our findings suggest that the potential for disease to spread quickly through the entire network increased with the amount of agricultural land and the connectivity of edge and decreased with the amount of forest. Other studies have found links between landscape structure and disease prevalence: Nobert et al. [32] found that landscape connectivity among known sites of CWD prevalence was an important predictor of risk. Likewise, Greer and Collins [87] showed that habitat configuration can affect the behavior of the host (Arizona tiger salamander, *Ambystoma tigrinum nebulosum*) and thus the incidence of disease (*A*. *tigrinum* virus).

It is possible that, had we been able to simultaneously monitor additional deer (i.e., larger social networks), we may have had more predictive power to uncover relationships between sociality and landscape characteristics. We note that our findings may not be generalizable beyond our study as deer behavior can vary regionally (e.g., [88], but see [62]). We attempted to control for the effect of uncollared deer on our estimates of deer sociality by using the average weighted degree rather than estimates that depend on how many neighbors were monitored, such as weighted degree or betweenness. Unless uncollared deer tended to be more (or less) social than collared deer, we do not expect that uncollared deer represented a bias. Finally, differences in deer population density among study areas may have impacted our findings. For example, variation in habitat suitability among study areas could result in a higher carrying capacity and, thus, higher population density in some areas. If the outcome of this was increased home range overlap, then variation in population density could have affected our estimates of sociality. The response of deer home range size to changes in population density, however, is unclear (e.g., [89–91]). Furthermore, population density was similar between the Carbondale and Shelbyville study areas.

Our work represents advancement in the general understanding of animal sociality, demonstrating that landscape structure can underlie both the local and global structure of social networks. Landscape structure can thus have implications for both individual and population fitness, as social network structure can influence the spread of infectious disease (e.g., [30,92]). Future research should test our predictions in areas where CWD is established: are deer in connected landscapes more likely to contract the disease, and is CWD prevalence higher in populations occurring where the proportion of agricultural land and the connectivity of edge are higher? Further, identifying the influence of landscape connectivity on the social behavior of male deer will be important because males tend to disperse farther [93,94] and are more susceptible to CWD infection [95,96]. Fine-scale movement path data will allow us to more closely assess the mechanisms driving the relationship between landscape connectivity and sociality, such as whether associations among animals occurred in connected landscapes, and the relative contributions of landcover connectivity and habitat-mediated fission-fusion dynamics on social behavior among deer groups. The degree of home range overlap is a strong predictor of association rates among deer; future research should address whether landscape connectivity influences sociality by affecting the spatial arrangement of home ranges. Finally, the interplay between extrinsic and intrinsic influences on animal sociality, such as identifying mechanisms driving the variation in an individual’s or a population’s behavioral response to landscape connectivity, will be an interesting avenue of future research.

## Supporting information

S1 Fig Mean association rate (95% confidence intervals in grey) for 8 female white-tailed deer (*Odocoileus virginianus*) dyads monitored simultaneously for an entire season. We randomly selected an increasing number of locations for each dyad, calculated association rate, and repeated this 100 times. The solid line represents the seasonal association rate (including all locations). The dashed lines represent the association rate that we would expect for a dyad with one more or one less association than what we observed for our focal dyad across the entire season; if the mean subsampled association rate was within these bounds, then the error associated with the number of locations should not interfere with our rank order of association rates. The value in the upper right corner is the number of locations at which the association rate was within the dashed lines.
(DOCX)

S2 Fig The difference between the bi-monthly association rate and the seasonal association rate for female white-tailed deer (*Odocoileus virginianus*) dyads during gestation (Jan 1 –May 14; n = 11), fawning (May 15 –Aug 31; n = 13), and the rut (Sep 1 –Dec 31; n = 13). Boxes represent the median (centre line) and the first and third quartiles (the range that contains 50% of the data). Whiskers represent the highest (lowest) value that is within 1.5*interquartile range. Points represent outliers and vertical lines delineate seasons. Data were pooled over study area (Carbondale and Lake Shelbyville) and year (2004–2009).
(DOCX)

S3 Fig The Carbondale study area (30km x 30km) with a 7.5km-wide buffer. The upper map is a conductance surface, where white represents forest (we assigned it a conductance value of 101) and black represents absence of forest (we assigned it a conductance value of 1). The lower map represents current density (white is high current and black is low current) across the forest conductance surface between pairs of sites (n = 50; white circles). The white box outlines the study area; the map outside of the box was later clipped to remove current density that was likely biased high due to the arbitrary placement of the 50 sites.
(DOCX)

S4 Fig Current density maps of the Carbondale, Lake Shelbyville, and Rend Lake study areas. White represents high current density of forest, agriculture, or the edge between forest and agriculture, and black represents low current density. Current density is analogous to random walkers moving across the landscape between sites located around the perimeter of the buffered study areas (S3 Fig).
(DOCX)

S5 Fig The relationship between standardized mean home range overlap and average edge weight for 36 female white-tailed deer (*Odocoileus virginianus*) in the Carbondale and Shelbyville study areas of Illinois, USA, pooled over season. We used a Box-Cox power transformation on the dependent variable (λ = 0.22;[53]) to reduce heteroscedasticity.
(DOCX)

S6 Fig Relationship between home range overlap and (log) association rate of 46 female white tailed deer (*Odocoileus virginianus*) in 7 networks between 1 Jan and 10 Mar (2005–2014) in Illinois, USA. We estimated home range overlap as the volume of intersection (VI) of 95% kernel utilization distributions [52] for each dyad during the time that both individuals were monitored. We estimated association rate as the number of times members of a dyad were within 25m of one another at the same time divided by the number of simultaneous locations. The 7 networks were from Carbondale 2005 (n = 6 deer) and 2012 (n = 6), Lake Shelbyville 2009 (n = 4), Crab Orchard 2014 (n = 5), Touch of Nature 2012 (n = 13) and 2013 (n- = 5), and Rend Lake 2014 (n = 4). We used the residuals of this relationship as edge weights in a social network.
(DOCX)

S7 Fig An example of a) the configuration of forest (white; conductance = 101) and non-forest (black; conductance = 1), and b) the corresponding current density of forest (high current density is white, low current density is black) in a portion of the Carbondale, IL study area. Areas of high current density (the probability of movement through a cell) tend to be where corridors of forest are surrounded by non-forest (i.e., pinch points).
(DOCX)

S1 Appendix Establishing rules for association rate data inclusion (DOCX)

S2 Appendix Fourteen linear models assessing seasonal relationships between female white-tailed deer (*Odocoileus virginianus*) sociality, indexed by average edge weight (edges weighted by association rate), and extrinsic factors at the local scale. (DOCX)

S3 Appendix Seasonal correlation among independent variables predicting female white-tailed deer (*Odocoileus virginianus*) sociality in southern Illinois, USA at the local scale. (DOCX)

S1 Table Landcover composition (%) of each study area in Illinois, USA. (DOCX)

S2 Table Mean seasonal home range size (km^2^) and home range overlap of female white tailed deer (*Odocoileus virginianus*) in 5 study areas in southern Illinois, USA. (DOCX)

S3 Table Seasonal association rates of female white-tailed deer (*Odocoileus virginianus*) in two study areas in central and southern Illinois, USA. (DOCX)

S4 Table Model-averaged coefficients (β), 95% confidence intervals (CI), and relative variable importance of standardized variables predicting average edge weight in networks of female white-tailed deer (*Odocoileus virginianus*) seasonal association rates in Carbondale, Illinois (2002–2006). Coefficients with 95% CI that did not overlap zero are in bold font.
(DOCX)

S5 Table Model-averaged coefficients (β), 95% confidence intervals (CI), and relative variable importance of standardized variables predicting average edge weight in networks of female white-tailed deer (*Odocoileus virginianus*) seasonal association rates in Lake Shelbyville, Illinois (2007–2009). Coefficients with 95% CI that did not overlap zero are in bold font.
(DOCX)
