# Supplementary material for: Does landscape connectivity shape local and global social network structure in white-tailed deer?
Source: PLoS One. 2017 Mar 17;12(3):e0173570. doi: 10.1371/journal.pone.0173570 (PMC5357016; doi:10.1371/journal.pone.0173570)
Supplement: S1 Fig — We randomly selected an increasing number of locations for each dyad, calculated association rate, and repeated this 100 times. The solid line represents the seasonal association rate (including all locations). The dashed lines represent the association rate that we would expect for a dyad with one more or one less association than what we observed for our focal dyad across the entire season; if the mean subsampled association rate was within these bounds, then the error associated with the number of locations should not interfere with our rank order of association rates. The value in the upper right corner is the number of locations at which the association rate was within the dashed lines. (DOCX) [file pone.0173570.s001.docx]

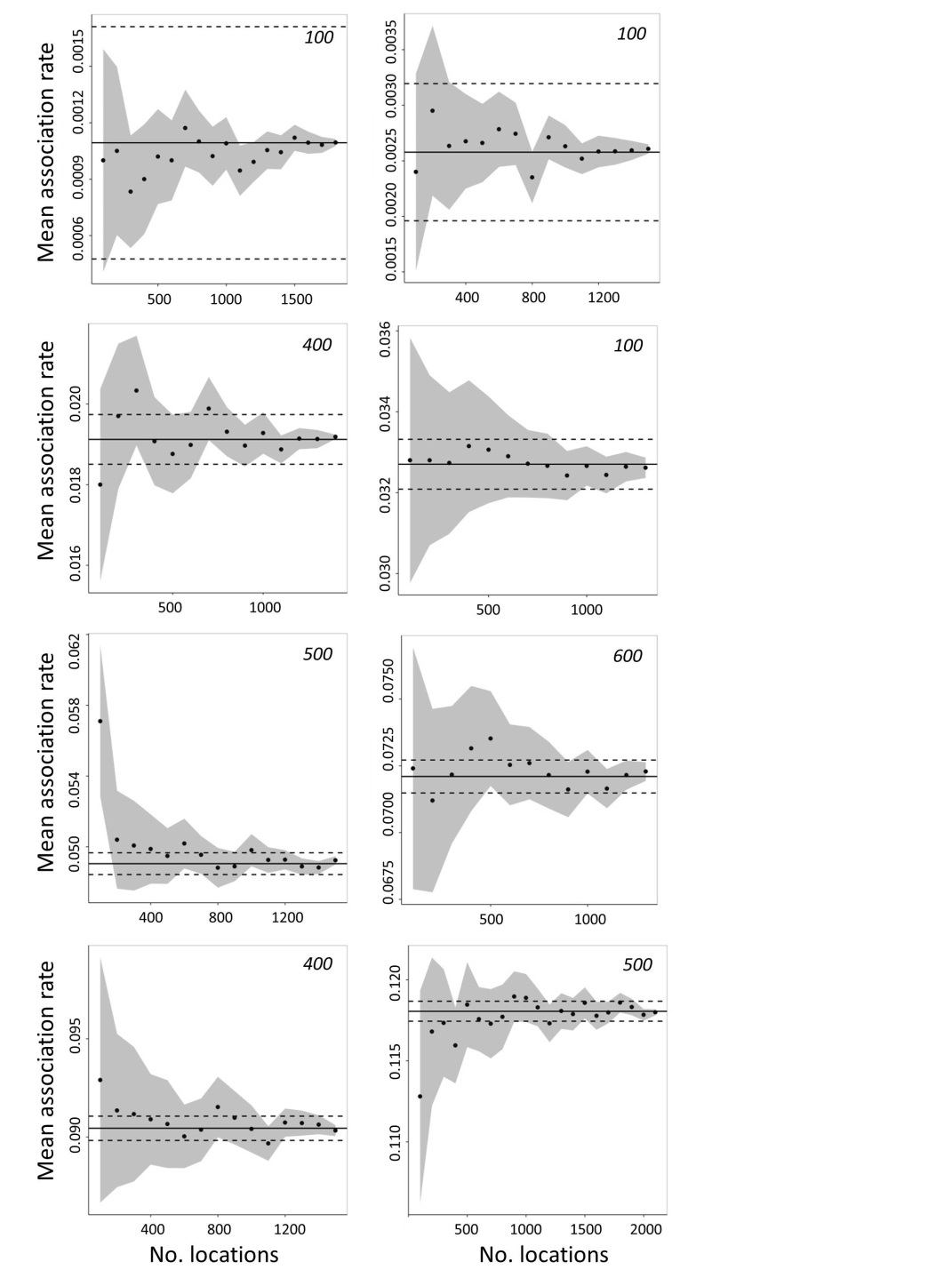


S1 Fig. Mean association rate (95% confidence intervals in grey) for 8 female white-tailed deer (*Odocoileus virginianus*) dyads monitored simultaneously for an entire season. We randomly selected an increasing number of locations for each dyad, calculated association rate, and repeated this 100 times. The solid line represents the seasonal association rate (including all locations). The dashed lines represent the association rate that we would expect for a dyad with one more or one less association than what we observed for our focal dyad across the entire season; if the mean subsampled association rate was within these bounds, then the error associated with the number of locations should not interfere with our rank order of association rates. The value in the upper right corner is the number of locations at which the association rate was within the dashed lines.
