# Supplementary material for: Does landscape connectivity shape local and global social network structure in white-tailed deer?
Source: PLoS One. 2017 Mar 17;12(3):e0173570. doi: 10.1371/journal.pone.0173570 (PMC5357016; doi:10.1371/journal.pone.0173570)
Supplement: S2 Fig — Boxes represent the median (centre line) and the first and third quartiles (the range that contains 50% of the data). Whiskers represent the highest (lowest) value that is within 1.5*interquartile range. Points represent outliers and vertical lines delineate seasons. Data were pooled over study area (Carbondale and Lake Shelbyville) and year (2004–2009). (DOCX) [file pone.0173570.s002.docx]

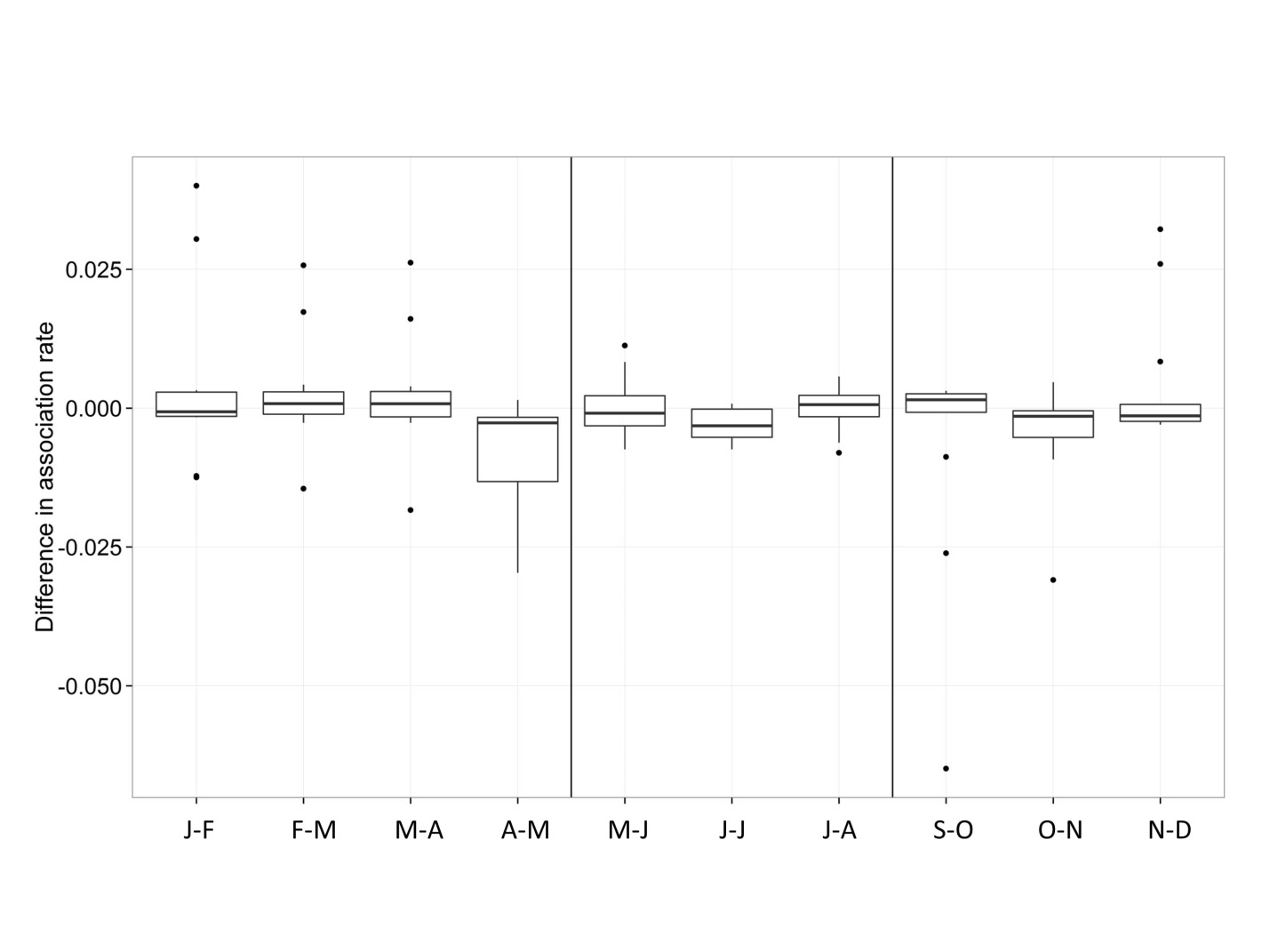


S2 Fig. The difference between the bi-monthly association rate and the seasonal association rate for female white-tailed deer (*Odocoileus virginianus*) dyads during gestation (Jan 1 – May 14; n = 11), fawning (May 15 – Aug 31; n = 13), and the rut (Sep 1 – Dec 31; n = 13). Boxes represent the median (centre line) and the first and third quartiles (the range that contains 50% of the data). Whiskers represent the highest (lowest) value that is within 1.5*interquartile range. Points represent outliers and vertical lines delineate seasons. Data were pooled over study area (Carbondale and Lake Shelbyville).
