# Supplementary material for: Does landscape connectivity shape local and global social network structure in white-tailed deer?
Source: PLoS One. 2017 Mar 17;12(3):e0173570. doi: 10.1371/journal.pone.0173570 (PMC5357016; doi:10.1371/journal.pone.0173570)
Supplement: S3 Fig — The upper map is a conductance surface, where white represents forest (we assigned it a conductance value of 101) and black represents absence of forest (we assigned it a conductance value of 1). The lower map represents current density (white is high current and black is low current) across the forest conductance surface between pairs of sites (n = 50; white circles). The white box outlines the study area; the map outside of the box was later clipped to remove current density that was likely biased high due to the arbitrary placement of the 50 sites. (DOCX) [file pone.0173570.s003.docx]

**
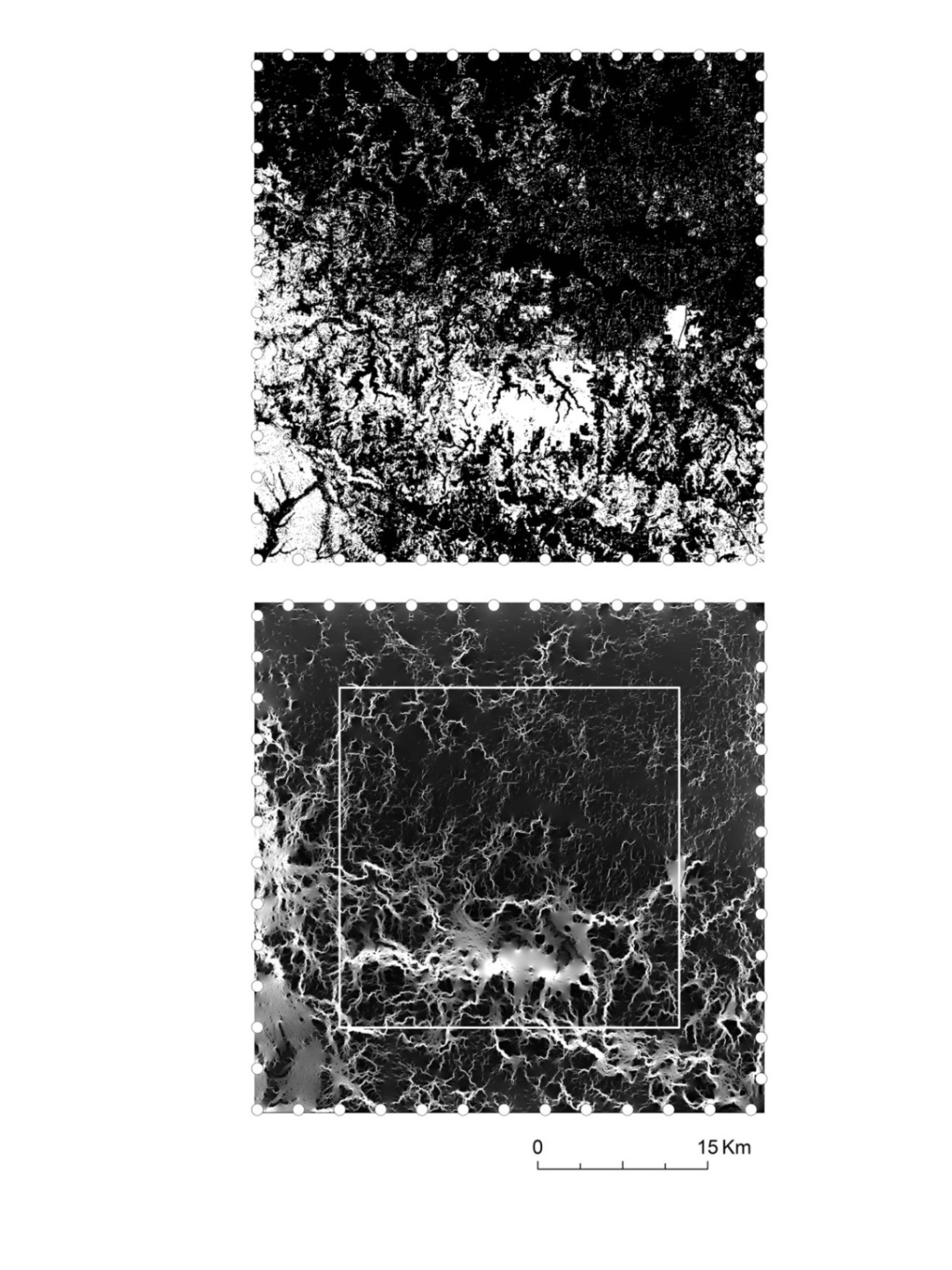
**

S3 Fig. The Carbondale study area (30km x 30km) with a 7.5km-wide buffer. The upper map is a conductance surface, where white represents forest (we assigned it a conductance value of 101) and black represents absence of forest (we assigned it a conductance value of 1). The lower map represents current density (white is high current and black is low current) across the forest conductance surface between pairs of sites (n=50; white circles). The white box outlines the study area; the map outside of the box was later clipped to remove current density that was likely biased high due to the arbitrary placement of the 50 sites.
