# Supplementary material for: Does landscape connectivity shape local and global social network structure in white-tailed deer?
Source: PLoS One. 2017 Mar 17;12(3):e0173570. doi: 10.1371/journal.pone.0173570 (PMC5357016; doi:10.1371/journal.pone.0173570)
Supplement: S4 Fig — White represents high current density of forest, agriculture, or the edge between forest and agriculture, and black represents low current density. Current density is analogous to random walkers moving across the landscape between sites located around the perimeter of the buffered study areas (S3 Fig). (DOCX) [file pone.0173570.s004.docx]

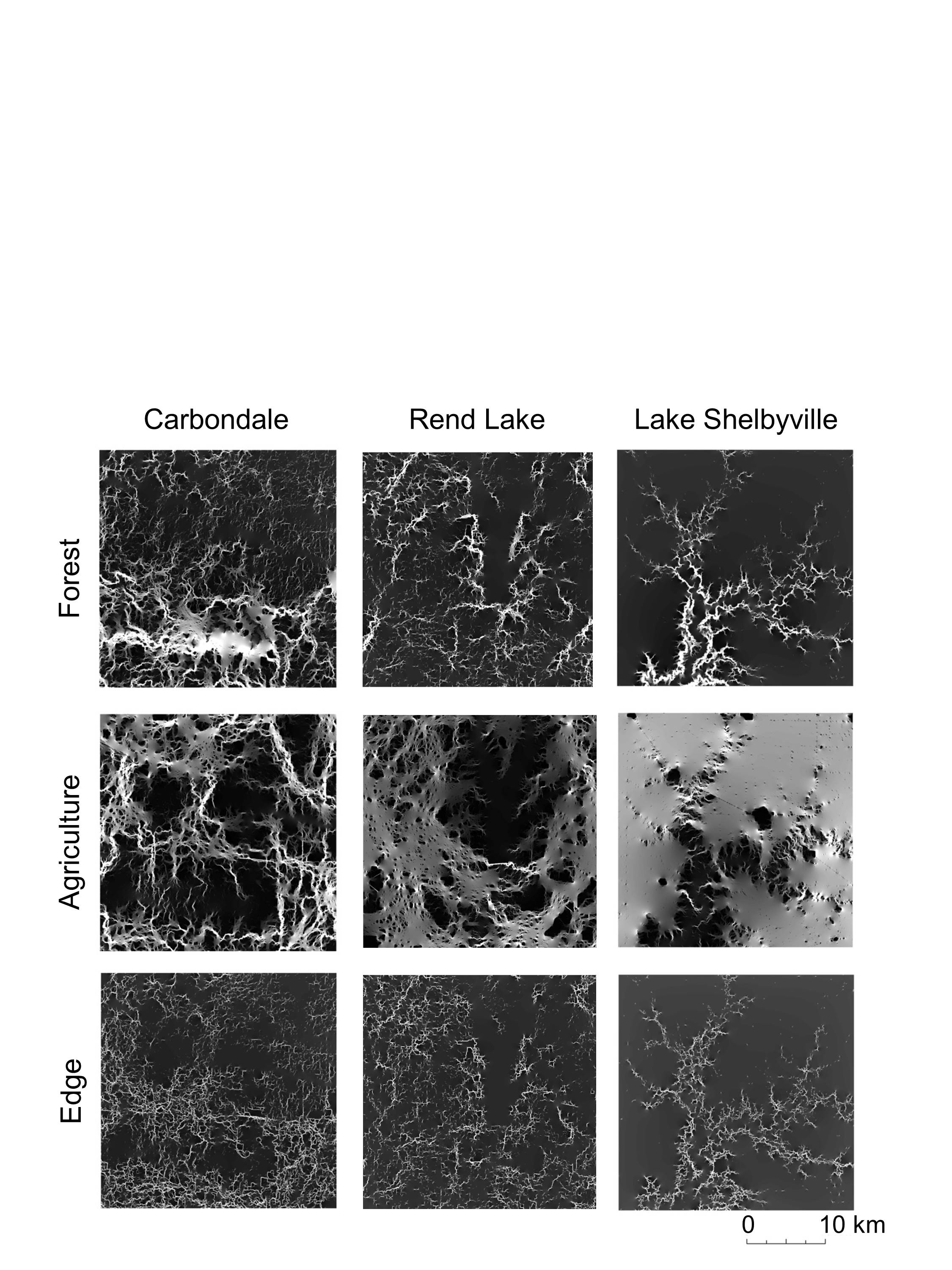


S4 Fig. Current density maps of the Carbondale, Lake Shelbyville, and Rend Lake study areas. White represents high current density of forest, agriculture, or the edge between forest and agriculture, and black represents low current density. Current density is analogous to random walkers moving across the landscape between sites located around the perimeter of the buffered study areas (S3 Fig).
