# Supplementary material for: Does landscape connectivity shape local and global social network structure in white-tailed deer?
Source: PLoS One. 2017 Mar 17;12(3):e0173570. doi: 10.1371/journal.pone.0173570 (PMC5357016; doi:10.1371/journal.pone.0173570)
Supplement: S5 Fig — We used a Box-Cox power transformation on the dependent variable (λ = 0.22;[53]) to reduce heteroscedasticity. (DOCX) [file pone.0173570.s005.docx]

S5 Fig. The relationship between standardized mean home range overlap and average edge weight for 36 female white-tailed deer (*Odocoileus virginianus*) in the Carbondale and Shelbyville study areas of Illinois, USA, pooled over season. We used a Box-Cox power transformation on the dependent variable (λ = 0.22; Box and Cox 1964) to reduce heteroscedasticity.

Box GE, Cox DR (1964) An analysis of transformations. J Roy Stat Soc B Met 26(2):211-252.
