# Supplementary material for: Does landscape connectivity shape local and global social network structure in white-tailed deer?
Source: PLoS One. 2017 Mar 17;12(3):e0173570. doi: 10.1371/journal.pone.0173570 (PMC5357016; doi:10.1371/journal.pone.0173570)
Supplement: S6 Fig — We estimated home range overlap as the volume of intersection (VI) of 95% kernel utilization distributions [52] for each dyad during the time that both individuals were monitored. We estimated association rate as the number of times members of a dyad were within 25m of one another at the same time divided by the number of simultaneous locations. The 7 networks were from Carbondale 2005 (n = 6 deer) and 2012 (n = 6), Lake Shelbyville 2009 (n = 4), Crab Orchard 2014 (n = 5), Touch of Nature 2012 (n = 13) and 2013 (n- = 5), and Rend Lake 2014 (n = 4). We used the residuals of this relationship as edge weights in a social network. (DOCX) [file pone.0173570.s006.docx]

S6 Fig. Relationship between home range overlap and (log) association rate of 46 female white tailed deer (*Odocoileus virginianus*) in 7 networks between 1 Jan and 10 Mar (2005-2014) in Illinois, USA. We estimated home range overlap as the volume of intersection (VI) of 95% kernel utilization distributions (Fieberg and Kochanny 2005) for each dyad during the time that both individuals were monitored. We estimated association rate as the number of times members of a dyad were within 25m of one another at the same time divided by the number of simultaneous locations. The 7 networks were from Carbondale 2005 (n=6 deer) and 2012 (n=6), Lake Shelbyville 2009 (n=4), Crab Orchard 2014 (n=5), Touch of Nature 2012 (n=13) and 2013 (n-=5), and Rend Lake 2014 (n=4). We used the residuals of this relationship as edge weights in a social network.

**References**

Fieberg, J., & Kochanny, C. O. (2005) Quantifying home-range overlap: the importance of the utilization distribution. Journal of Wildlife Management, 69(4), 1346-1359.
