# Supplementary material for: Does landscape connectivity shape local and global social network structure in white-tailed deer?
Source: PLoS One. 2017 Mar 17;12(3):e0173570. doi: 10.1371/journal.pone.0173570 (PMC5357016; doi:10.1371/journal.pone.0173570)
Supplement: S7 Fig — An example of a) the configuration of forest (white; conductance = 101) and non-forest (black; conductance = 1), and b) the corresponding current density of forest (high current density is white, low current density is black) in a portion of the Carbondale, IL study area. Areas of high current density (the probability of movement through a cell) tend to be where corridors of forest are surrounded by non-forest (i.e., pinch points). (DOCX) [file pone.0173570.s007.docx]

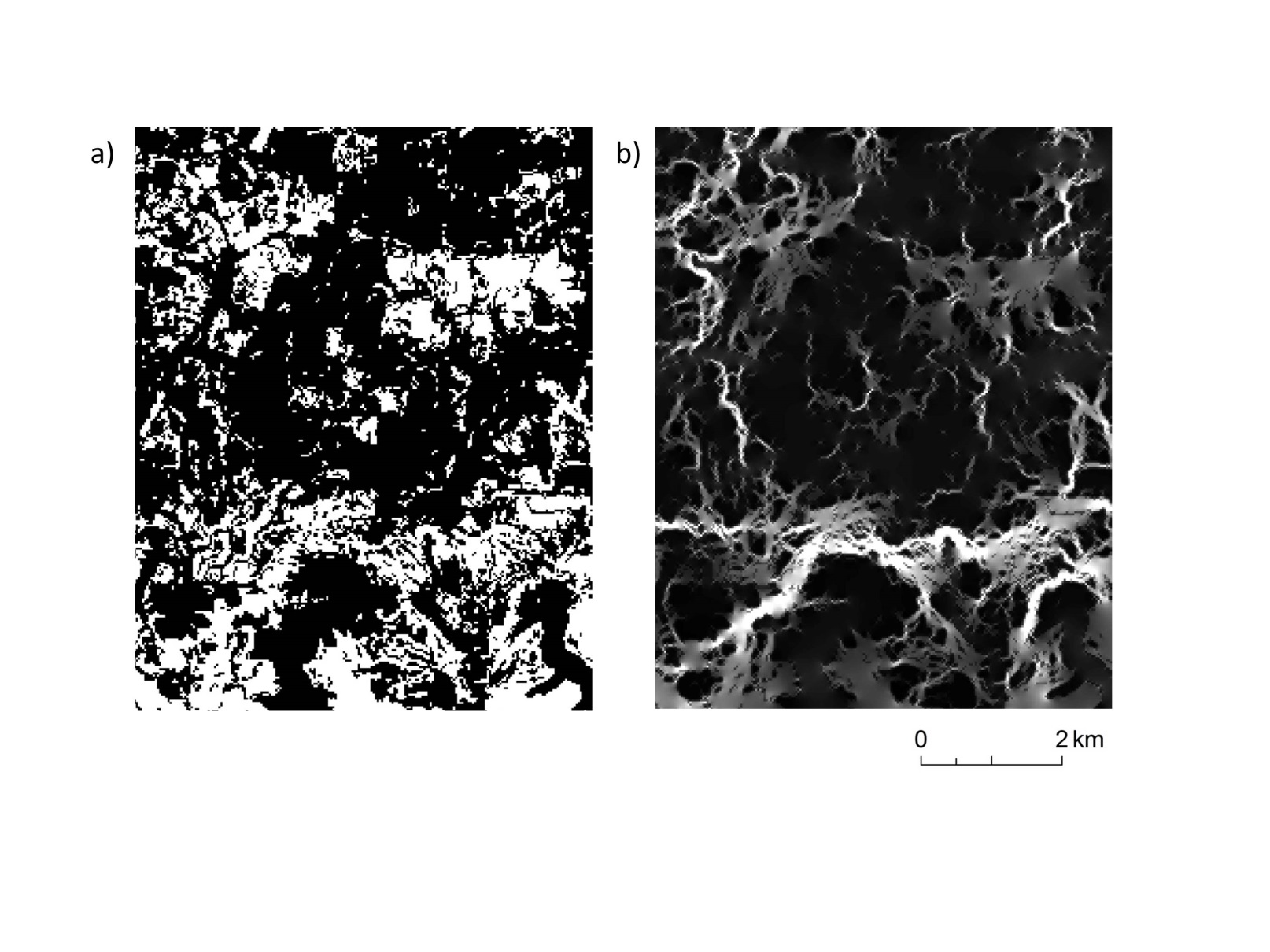


S7 Fig. An example of a) the configuration of forest (white; conductance = 101) and non-forest (black; conductance = 1), and b) the corresponding current density of forest (high current density is white, low current density is black) in a portion of the Carbondale, IL study area. Areas of high current density (the probability of movement through a cell) tend to be where corridors of forest are surrounded by non-forest (i.e., pinch points).
