# Supplementary material for: Does landscape connectivity shape local and global social network structure in white-tailed deer?
Source: PLoS One. 2017 Mar 17;12(3):e0173570. doi: 10.1371/journal.pone.0173570 (PMC5357016; doi:10.1371/journal.pone.0173570)
Supplement: S1 Appendix — (DOCX) [file pone.0173570.s008.docx]

**S1 Appendix. Establishing rules for association rate data inclusion**

**a. Number of simultaneous locations**

We sought to identify the minimum number of simultaneous locations necessary to obtain an accurate estimate of the seasonal association rate. From the Carbondale (2002-2006) and Lake Shelbyville (2007-2009) datasets, we selected a set of 8 focal dyads with a range of seasonal association rates (0.001 – 0.118) that were monitored for an entire season. From each dyad, we randomly chose *k* simultaneous locations in multiples of 100, calculated the association rate, and repeated this 100 times. In particular, we wanted to find the number of locations that ensured that the relative error (i.e., the difference between the mean (over 100 replicates) and seasonal association rates) was not larger than the signal (i.e., the lowest expected association rate). To estimate the lowest expected association rate, we calculated the maximum possible number of simultaneous locations for a dyad in one season (135 days * 12 locations/day) and assumed that two deer came within 25m of each other at the same time only once. This would result in an association rate of 0.000617 and represents the smallest association rate that we could expect. This value is conservative because many dyads had fewer than 1620 simultaneous locations.

We plotted the mean association rate as the number of locations increased in relation to the association rate that we would expect for a dyad with one more or one less association (S1 Fig.). We found that the relative error of association rate was within an acceptable range (i.e., |average association rate| < 0.000617) when we used >600 locations (S1 Fig.). Thus, at least 600 simultaneous locations per dyad should ensure that our rank order of association rates across dyads was not influenced by the number of simultaneous locations.

**b. Bi-monthly association rates**

We were interested in identifying months with association rates of female white-tailed deer (*Odocoileus virginianus*) that differed from the seasonal association rate. First, we selected all pairs of deer collared for an entire season that had a seasonal association rate >0 from the Carbondale (2002-2006) and Lake Shelbyville (2007-2009) datasets only. Pooled over study area and year, this yielded 11 dyads during gestation (Jan 1 – May 14), 13 dyads during fawning (May 15 – Aug 31), and 13 dyads during the rut (Sep 1 – Dec 31). These data were not independent; several animals belonged to more than one dyad in a season, and several dyads were monitored over >1 season.

For each dyad, we calculated the difference between the bi-monthly and the seasonal association rate (S2 Fig.). We used this information to construct rules for data inclusion by assuming that 2-month intervals with association rates that were not different than the seasonal rate were representative of the entire season. For example, if association rates for Feb-Mar were similar to the seasonal rate, and a dyad was collared simultaneously for Feb and Mar only, we assumed that the Feb-Mar association rate for this dyad was representative of the entire gestation season, and we included it in our analysis. If, however, association rates for Apr-May were lower than the seasonal association rate and a dyad was monitored for April and May only, we did not include this dyad in our analysis because it is likely that the association rate was biased low and not representative of the gestation season.

This difference between bi-monthly and seasonal association rates was significantly lower than zero for Apr-May (mean (SD) difference = -0.008 (0.010), p = 0.023) and Jun-Jul (mean (SD) difference = -0.002 (0.003), p = 0.006). For all other 2-month intervals, the bi-monthly association rate was not different than the seasonal rate (P>0.100; S2 Fig.). Therefore, we established the following rules for inclusion of dyads that were monitored simultaneously for less than any full season: 1) all dyads must have >600 simultaneous locations; and 2) simultaneous locations must not occur in Apr-May only or Jun-Jul only.
