# Supplementary material for: Does landscape connectivity shape local and global social network structure in white-tailed deer?
Source: PLoS One. 2017 Mar 17;12(3):e0173570. doi: 10.1371/journal.pone.0173570 (PMC5357016; doi:10.1371/journal.pone.0173570)
Supplement: S2 Appendix — (DOCX) [file pone.0173570.s009.docx]

**S2 Appendix.** Fourteen linear models assessing seasonal relationships between female white-tailed deer (*Odocoileus virginianus*) sociality, indexed by average edge weight (edges weighted by association rate), and extrinsic factors at the local scale. For each individual, we calculated the proportion of each landcover type within the seasonal home range and connectivity of each landcover type as the mean current density within the seasonal home range. We calculated home range overlap as the mean probability of a neighboring deer being within an individual’s 95% kernel density home range during the time that each pair of deer was simultaneously monitored. We did not include multiple landcover variables in the same model because of multicollinearity (S3 appendix).

ave. edge weight^0.22^ = 1

ave. edge weight^0.22^  = home range overlap

ave. edge weight^0.22^  = forest proportion

ave. edge weight^0.22^  = forest connectivity

ave. edge weight^0.22^  = agriculture proportion

ave. edge weight^0.22^  = agriculture connectivity

ave. edge weight^0.22^  = edge proportion

ave. edge weight^0.22^  = edge connectivity

ave. edge weight^0.22^  = forest proportion + home range overlap

ave. edge weight^0.22^  = forest connectivity + home range overlap

ave. edge weight^0.22^  = agriculture proportion + home range overlap

ave. edge weight^0.22^  = agriculture connectivity + home range overlap

ave. edge weight^0.22^  = edge proportion + home range overlap

ave. edge weight^0.22^  = edge connectivity + home range overlap
