# Supplementary material for: Does landscape connectivity shape local and global social network structure in white-tailed deer?
Source: PLoS One. 2017 Mar 17;12(3):e0173570. doi: 10.1371/journal.pone.0173570 (PMC5357016; doi:10.1371/journal.pone.0173570)
Supplement: S3 Appendix — (DOCX) [file pone.0173570.s010.docx]

**S3 Appendix.** Seasonal correlation among independent variables predicting female white-tailed deer (*Odocoileus virginianus*) sociality in southern Illinois, USA at the local scale. We obtained landcover data from the Illinois Natural History Survey Illinois Gap Analysis Land Cover Classification from 1999 and 2000 (INHS 2003). We pooled data over the Carbondale and Lake Shelbyville study areas.

**References**

INHS (2003) Illinois Natural History Survey's 1999-2000 1:100 000 Scale Illinois Gap Analysis Land Cover Classification, Raster Digital Data, Version 2.0, September 2003.

Pearson correlation coefficients (*r*) among independent variables, based on the mean landcover proportion (lower) or connectivity (current density; upper) within each individual’s home range (n=51) during the **gestation period** (Jan 1 – May 14) at the local scale. Coefficients of correlation between landcover amount and connectivity are in italics along the diagonal. Coefficients >|0.5| are in bold font.

|  | Forest | Agriculture | Edge | Home range overlap |
| --- | --- | --- | --- | --- |
| Forest | *-0.05* | **-0.80** | 0.44 | 0.25 |
| Agriculture | **-0.68** | ***0.67*** | **-0.50** | -0.15 |
| Edge | **0.59** | -0.06 | ***0.50*** | -0.16 |
| Home range overlap | 0.17 | -0.29 | -0.12 |  |

Pearson correlation coefficients (*r*) among independent variables, based on the mean landcover proportion (lower) or connectivity (current density; upper) within each individual’s home range (n=21) during the **fawning period** (May 15 – Aug 31) at the local scale. Coefficients of correlation between landcover amount and connectivity are in italics along the diagonal. Coefficients >|0.5| are in bold font.

|  | Forest | Agriculture | Edge | Home range overlap |
| --- | --- | --- | --- | --- |
| Forest | *0.22* | **-0.69** | **0.57** | -0.05 |
| Agriculture | **-0.80** | ***0.64*** | **-0.55** | -0.15 |
| Edge | 0.40 | -0.01 | *0.49* | -0.19 |
| Home range overlap | -0.32 | 0.06 | -0.48 |  |

Pearson correlation coefficients (*r*) among independent variables, based on the mean landcover proportion (lower) or connectivity (current density; upper) within each individual’s home range (n=25) during the **rut** (Sep 1 - Dec 31) at the local scale. Coefficients of correlation between landcover amount and connectivity are in italics along the diagonal. Coefficients >|0.5| are in bold font.

|  | Forest | Agriculture | Edge | Home range overlap |
| --- | --- | --- | --- | --- |
| Forest | *0.10* | **-0.75** | **0.56** | **0.53** |
| Agriculture | **-0.65** | ***0.77*** | -0.25 | -0.41 |
| Edge | 0.31 | 0.31 | *0.47* | 0.25 |
| Home range overlap | -0.06 | -0.35 | -0.42 |  |
