# Supplementary material for: Does landscape connectivity shape local and global social network structure in white-tailed deer?
Source: PLoS One. 2017 Mar 17;12(3):e0173570. doi: 10.1371/journal.pone.0173570 (PMC5357016; doi:10.1371/journal.pone.0173570)
Supplement: S1 Table — (DOCX) [file pone.0173570.s011.docx]

S1 Table. Landcover composition^a^ (%) of each study area in Illinois, USA.

| Study area | Corn | Soy | Rural grassland | Forest | Urban | Wetland | Water | Edge^b^ |
| --- | --- | --- | --- | --- | --- | --- | --- | --- |
| Carbondale | 7 | 12 | 28 | 29 | 4 | 6 | 11 | 14 |
| Lake Shelbyville | 25 | 24 | 13 | 12 | 1 | 8 | 16 | 6 |
| Crab Orchard | 1 | 1 | 11 | 64 | <1 | 3 | 18 | 10 |
| Touch of Nature | 1 | 1 | 12 | 66 | <1 | 3 | 15 | 11 |
| Rend Lake | 3 | 6 | 18 | 18 | 1 | 7 | 42 | 9 |

^a^ Landcover data were from the Illinois Natural History Survey Illinois Gap Analysis Land Cover Classification from 1999 and 2000 (INHS 2003). Forest represented dry, mesic, and dry-mesic upland forest, and mesic and wet-mesic floodplain forest. The rural grassland category consisted of permanent pastureland, roadsides and fence lines, railroad right-of-ways, waterways, prairies, and other grassland cover. Water represented lakes and rivers, and wetlands included treed and untreed wetlands. We calculated the proportion of each landcover type within a 3km radius of the centroid of each study area. We defined the centroid of the study area as the centroid of a 100% minimum convex polygon around all locations.

^b^ We used Geospatial Modelling Environment software (version 0.7.2.0; Beyer 2012) to find the edges between agriculture and forest and then coded 30m x 30m grid cells based on presence or absence of edge. Values represent the percent area within a 3km radius of the study area centroid designated as edge (relative to non-edge).

**References**

Beyer HL (2012) Geospatial Modelling Environment (Version 0.7.2.0). http://www.spatialecology.com/gme.

INHS (2003) Illinois Natural History Survey's 1999-2000 1:100 000 Scale Illinois Gap Analysis Land Cover Classification, Raster Digital Data, Version 2.0, September 2003.
