# Supplementary material for: Does landscape connectivity shape local and global social network structure in white-tailed deer?
Source: PLoS One. 2017 Mar 17;12(3):e0173570. doi: 10.1371/journal.pone.0173570 (PMC5357016; doi:10.1371/journal.pone.0173570)
Supplement: S2 Table — (DOCX) [file pone.0173570.s012.docx]

**S2 Table.** Mean seasonal home range size (km^2^) and home range overlap of female white tailed deer (*Odocoileus virginianus*) in 5 study areas in southern Illinois, USA.

| Season^a^ | Study area | Home range size^b^ | | | Home range overlap^c^ | | |
| --- | --- | --- | --- | --- | --- | --- | --- |
|  |  | Mean | SD | No. deer | Mean | SD | No. dyads |
| Gestation | Carbondale (2003-06) | 1.27 | 0.48 | 25 | 0.22 | 0.19 | 32 |
|  | Carbondale (2012) | 0.60 | 0.21 | 7 | 0.17 | 0.17 | 15 |
|  | Lake Shelbyville (2007-09) | 2.23 | 1.95 | 21 | 0.19 | 0.17 | 11 |
|  | Crab Orchard (2014) | 1.64 | 0.81 | 6 | 0.25 | 0.22 | 10 |
|  | Touch of Nature (2012-13) | 0.50 | 0.19 | 16 | 0.14 | 0.18 | 54 |
|  | Rend Lake (2014) | 1.00 | 0.37 | 10 | 0.47 | 0.15 | 6 |
| Fawning | Carbondale (2003-05) | 1.21 | 1.03 | 20 | 0.11 | 0.11 | 21 |
|  | Lake Shelbyville (2006-08) | 0.91 | 0.65 | 18 | 0.21 | 0.23 | 9 |
| Rut | Carbondale (2002-05) | 1.53 | 3.42 | 19 | 0.18 | 0.19 | 34 |
|  | Lake Shelbyville (2006-08) | 1.45 | 1.07 | 18 | 0.40 | 0.12 | 7 |

^a^ Gestation (1 Jan – 10 Mar), fawning (15 May – 31 Aug), rut (1 Sep – 31 Dec).

^b^  95% kernel density estimate(KDE) for animals with at least 600 locations.

^c^ Volume of home range (KDE) intersection for deer dyads during the time that they were both monitored (VI; Fieberg J, Kochanny CO. Quantifying home-range overlap: the importance of the utilization distribution. J Wildlife Manage. 2005;69(4): 1346-1359). Averages include only dyads with >600 simultaneous locations and >0 home range overlap. Values range from 0 (no overlap) to 1 (complete overlap).
