# Supplementary material for: Does landscape connectivity shape local and global social network structure in white-tailed deer?
Source: PLoS One. 2017 Mar 17;12(3):e0173570. doi: 10.1371/journal.pone.0173570 (PMC5357016; doi:10.1371/journal.pone.0173570)
Supplement: S3 Table — (DOCX) [file pone.0173570.s013.docx]

S3 Table. Seasonal association rates^a^ of female white-tailed deer (*Odocoileus virginianus*) in two study areas^b^ in central and southern Illinois, USA.

| Study area | Season^c^ | Mean | SD | Min | Max | No. dyads |
| --- | --- | --- | --- | --- | --- | --- |
| Carbondale | Gestation | 0.0104 | 0.0217 | 0 | 0.1190 | 52 |
|  | Fawning | 0.0050 | 0.0098 | 0 | 0.0321 | 21 |
|  | Rut | 0.0086 | 0.0207 | 0 | 0.1030 | 34 |
| Lake Shelbyville | Gestation | 0.0109 | 0.0247 | 0 | 0.0967 | 32 |
|  | Fawning | 0.0066 | 0.0067 | 0 | 0.0219 | 9 |
|  | Rut | 0.0132 | 0.0156 | 0 | 0.0463 | 7 |

^a^ The number of times two female white-tailed deer were within 25m of one another at the same time (within 3 minutes), divided by the total number of simultaneous locations. Association rates were based on dyads with >600 simultaneous locations and home range overlap >0 during the time that both animals were monitored.

^b^ 24 female deer were collared between 2002 and 2006 in the Carbondale study area, and 12 female deer were collared between 2007 and 2009 in the Lake Shelbyville study area.

^c^ Gestation (1 Jan – 14 May), fawning (15 May – 31 Aug), rut (1 Sep – 31 Dec).
