# Supplementary material for: Does landscape connectivity shape local and global social network structure in white-tailed deer?
Source: PLoS One. 2017 Mar 17;12(3):e0173570. doi: 10.1371/journal.pone.0173570 (PMC5357016; doi:10.1371/journal.pone.0173570)
Supplement: S4 Table — Coefficients with 95% CI that did not overlap zero are in bold font. (DOCX) [file pone.0173570.s014.docx]

S4 Table. Model-averaged coefficients (β)^a^, 95% confidence intervals (CI), and relative variable importance of standardized variables predicting average edge weight in networks of female white-tailed deer (*Odocoileus virginianus*) seasonal association rates in Carbondale, Illinois (2002-2006). Coefficients with 95% CI that did not overlap zero are in bold font.

| Season^b^ | Variable^c^ | β | 95% CI | | Variable importance^d^ |
| --- | --- | --- | --- | --- | --- |
|  |  |  | Lower | Upper |  |
| Gestation | Intercept | 0.2817 | 0.2509 | 0.3126 |  |
|  | **HR overlap** | **0.1039** | **0.0723** | **0.1356** |  |
|  | Forest (conn) | -0.0241 | -0.0549 | 0.0067 | 0.21 |
|  | Edge (conn) | -0.0223 | -0.0535 | 0.0089 | 0.17 |
|  | Edge (prop) | -0.0179 | -0.0495 | 0.0138 | 0.12 |
|  | Ag (conn) | 0.0145 | -0.0179 | 0.0470 | 0.09 |
|  | Ag (prop) | 0.0141 | -0.0178 | 0.0460 | 0.09 |
|  | Forest (prop) | -0.0088 | -0.0412 | 0.0235 | 0.07 |
|  |  |  |  |  |  |
| Fawning | Intercept | 0.2589 | 0.2233 | 0.2944 |  |
|  | **Forest (conn)** | **-0.0882** | **-0.1235** | **-0.0530** | **0.80** |
|  | **Forest (prop)** | **-0.0818** | **-0.1235** | **-0.0401** | **0.14** |
|  | **Ag (conn)** | **0.0730** | **0.0248** | **0.1213** | **0.03** |
|  | **Ag (prop)** | **0.0762** | **0.0265** | **0.1258** | **0.03** |
|  | HR overlap | 0.0288 | -0.0074 | 0.0649 |  |
|  |  |  |  |  |  |
| Rut | Intercept | 0.3055 | 0.2586 | 0.3525 |  |
|  | **HR overlap** | **0.0650** | **0.0168** | **0.1132** |  |
|  | Forest (conn) | -0.0138 | -0.0698 | 0.0421 | 0.10 |
|  | Ag (prop) | 0.0124 | -0.0395 | 0.0644 | 0.10 |
|  | Ag (conn) | 0.0133 | -0.0359 | 0.0625 | 0.10 |
|  | Edge (prop) | 0.0083 | -0.0413 | 0.0578 | 0.09 |
|  | Forest (prop) | -0.0035 | -0.0566 | 0.0495 | 0.09 |
|  | Edge (conn) | 0.0037 | -0.0466 | 0.0539 | 0.08 |

^a^ Averaged over all models (Supplement 2). We present only variables that appeared in the top models (Δ AICc < 8; Table 1).

^b^ Gestation (1 Jan – 14 May; n = 24), fawning (15 May – 31 Aug; n = 11), rut (1 Sep – 31 Dec; n = 17)

^c^ Variables are described in the footnote of Table 1.

^d^ We did not include importance values for home range overlap because it was present in 7 models, whereas the landcover variables were each present in 2 models.
