# Supplementary material for: Does landscape connectivity shape local and global social network structure in white-tailed deer?
Source: PLoS One. 2017 Mar 17;12(3):e0173570. doi: 10.1371/journal.pone.0173570 (PMC5357016; doi:10.1371/journal.pone.0173570)
Supplement: S5 Table — Coefficients with 95% CI that did not overlap zero are in bold font. (DOCX) [file pone.0173570.s015.docx]

S5 Table. Model-averaged coefficients (β)^a^, 95% confidence intervals (CI), and relative variable importance of standardized variables predicting average edge weight in networks of female white-tailed deer (*Odocoileus virginianus*) seasonal association rates in Lake Shelbyville, Illinois (2007-2009). Coefficients with 95% CI that did not overlap zero are in bold font.

| Season^b^ | Variable^c^ | β | 95% CI | | Variable importance^d^ |
| --- | --- | --- | --- | --- | --- |
|  |  |  | Lower | Upper |  |
| Gestation | (Intercept) | 0.2506 | 0.1943 | 0.3068 |  |
|  | **HR overlap** | **0.1352** | **0.0723** | **0.1981** |  |
|  | Ag (prop) | 0.0234 | -0.0402 | 0.0870 | 0.08 |
|  | Ag (conn) | 0.0160 | -0.0695 | 0.1015 | 0.07 |
|  | Forest (conn) | 0.0102 | -0.0512 | 0.0715 | 0.06 |
|  | Forest (prop) | 0.0087 | -0.0524 | 0.0697 | 0.06 |
|  | Edge (prop) | -0.0095 | -0.0962 | 0.0771 | 0.06 |
|  | Edge (conn) | -0.0085 | -0.1100 | 0.0930 | 0.06 |
|  |  |  |  |  |  |
| Fawning | (Intercept) | 0.2293 | 0.1318 | 0.3267 |  |
|  | HR overlap | 0.0839 | -0.0162 | 0.1840 |  |
|  | Ag (conn) | -0.0289 | -0.1350 | 0.0772 | 0.07 |
|  | Ag (prop) | -0.0247 | -0.1328 | 0.0834 | 0.07 |
|  | Edge (prop) | -0.0064 | -0.1237 | 0.1108 | 0.06 |
|  | Forest (prop) | 0.0280 | -0.0844 | 0.1405 | 0.07 |
|  | Edge (conn) | 0.0039 | -0.1162 | 0.1241 | 0.06 |
|  | Forest (conn) | 0.0141 | -0.0993 | 0.1275 | 0.06 |
|  |  |  |  |  |  |
| Rut | (Intercept) | 0.3945 | 0.3414 | 0.4476 |  |
|  | **Ag (prop)** | **0.0839** | **0.0456** | **0.1222** | **0.40** |
|  | **Ag (conn)** | **0.0820** | **0.0400** | **0.1240** | **0.23** |
|  | Edge (conn) | 0.0635 | -0.0025 | 0.1295 | 0.02 |
|  | Edge (prop) | 0.0542 | -0.0193 | 0.1277 | 0.01 |
|  | Forest (conn) | -0.0527 | -0.1273 | 0.0218 | 0.01 |

^a^ Averaged over all models (Supplement 2). We present only variables that appeared in the top models (Δ AICc < 8; Table 2).

^b^ Gestation (1 Jan – 14 May; n = 12), fawning (15 May – 31 Aug; n = 10), rut (1 Sep – 31 Dec; n = 6)

^c^ Variables are described in the footnote of Table 1.

^d^ We did not include importance values for home range overlap because it was present in 7 models, whereas the landcover variables were each present in 2 models.
